# COVID-19 Cases and Hospitalizations by COVID-19 Vaccination Status and Previous COVID-19 Diagnosis — California and New York, May–November 2021

**DOI:** 10.15585/mmwr.mm7104e1

**Published:** 2022-01-28

**Authors:** Tomás M. León, Vajeera Dorabawila, Lauren Nelson, Emily Lutterloh, Ursula E. Bauer, Bryon Backenson, Mary T. Bassett, Hannah Henry, Brooke Bregman, Claire M. Midgley, Jennifer F. Myers, Ian D. Plumb, Heather E. Reese, Rui Zhao, Melissa Briggs-Hagen, Dina Hoefer, James P. Watt, Benjamin J. Silk, Seema Jain, Eli S. Rosenberg

**Affiliations:** ^1^California Department of Public Health; ^2^New York State Department of Health; ^3^University at Albany School of Public Health, SUNY, Rensselaer, New York; ^4^CDC.

By November 30, 2021, approximately 130,781 COVID-19–associated deaths, one in six of all U.S. deaths from COVID-19, had occurred in California and New York.* COVID-19 vaccination protects against infection with SARS-CoV-2 (the virus that causes COVID-19), associated severe illness, and death (*1*,*2*); among those who survive, previous SARS-CoV-2 infection also confers protection against severe outcomes in the event of reinfection (*3*,*4*). The relative magnitude and duration of infection- and vaccine-derived protection, alone and together, can guide public health planning and epidemic forecasting. To examine the impact of primary COVID-19 vaccination and previous SARS-CoV-2 infection on COVID-19 incidence and hospitalization rates, statewide testing, surveillance, and COVID-19 immunization data from California and New York (which account for 18% of the U.S. population) were analyzed. Four cohorts of adults aged ≥18 years were considered: persons who were 1) unvaccinated with no previous laboratory-confirmed COVID-19 diagnosis, 2) vaccinated (14 days after completion of a primary COVID-19 vaccination series) with no previous COVID-19 diagnosis, 3) unvaccinated with a previous COVID-19 diagnosis, and 4) vaccinated with a previous COVID-19 diagnosis. Age-adjusted hazard rates of incident laboratory-confirmed COVID-19 cases in both states were compared among cohorts, and in California, hospitalizations during May 30–November 20, 2021, were also compared. During the study period, COVID-19 incidence in both states was highest among unvaccinated persons without a previous COVID-19 diagnosis compared with that among the other three groups. During the week beginning May 30, 2021, compared with COVID-19 case rates among unvaccinated persons without a previous COVID-19 diagnosis, COVID-19 case rates were 19.9-fold (California) and 18.4-fold (New York) lower among vaccinated persons without a previous diagnosis; 7.2-fold (California) and 9.9-fold lower (New York) among unvaccinated persons with a previous COVID-19 diagnosis; and 9.6-fold (California) and 8.5-fold lower (New York) among vaccinated persons with a previous COVID-19 diagnosis. During the same period, compared with hospitalization rates among unvaccinated persons without a previous COVID-19 diagnosis, hospitalization rates in California followed a similar pattern. These relationships changed after the SARS-CoV-2 Delta variant became predominant (i.e., accounted for >50% of sequenced isolates) in late June and July. By the week beginning October 3, compared with COVID-19 cases rates among unvaccinated persons without a previous COVID-19 diagnosis, case rates among vaccinated persons without a previous COVID-19 diagnosis were 6.2-fold (California) and 4.5-fold (New York) lower; rates were substantially lower among both groups with previous COVID-19 diagnoses, including 29.0-fold (California) and 14.7-fold lower (New York) among unvaccinated persons with a previous diagnosis, and 32.5-fold (California) and 19.8-fold lower (New York) among vaccinated persons with a previous diagnosis of COVID-19. During the same period, compared with hospitalization rates among unvaccinated persons without a previous COVID-19 diagnosis, hospitalization rates in California followed a similar pattern. These results demonstrate that vaccination protects against COVID-19 and related hospitalization, and that surviving a previous infection protects against a reinfection and related hospitalization. Importantly, infection-derived protection was higher after the Delta variant became predominant, a time when vaccine-induced immunity for many persons declined because of immune evasion and immunologic waning (*2*,*5*,*6*). Similar cohort data accounting for booster doses needs to be assessed, as new variants, including Omicron, circulate. Although the epidemiology of COVID-19 might change with the emergence of new variants, vaccination remains the safest strategy to prevent SARS-CoV-2 infections and associated complications; all eligible persons should be up to date with COVID-19 vaccination. Additional recommendations for vaccine doses might be warranted in the future as the virus and immunity levels change.

Four cohorts of persons aged ≥18 years were assembled via linkages of records from electronic laboratory reporting databases and state-specific immunization information systems.^†^ Persons were classified based on whether they had had a laboratory-confirmed SARS-CoV-2 infection by March 1, 2021 (i.e., previous COVID-19 diagnosis)^§^; had received at least the primary COVID-19 vaccination series^¶^ by May 16, 2021; had a previous COVID-19 diagnosis and were fully vaccinated**; or had neither received a previous COVID-19 diagnosis by March 1 nor received a first COVID-19 vaccine dose by the end of the analysis period. The size of the unvaccinated group without a previous diagnosis was derived by subtracting the observed groups from U.S. Census estimates.^††^ To maintain each defined cohort, persons who received a COVID-19 diagnosis during March 1–May 30, 2021, or who died before May 30, 2021, were excluded (to maintain eligibility for incident cases for all cohorts on May 30, 2021),^§§^ as were persons who received a first vaccine dose during May 30–November 20, 2021. During May 30–November 20, 2021, incident cases were defined using a positive nucleic acid amplification test (NAAT) result from the California COVID-19 Reporting System (CCRS) or a positive NAAT or antigen test result from the New York Electronic Clinical Laboratory Reporting System. In California, person-level hospitalization data from CCRS and supplementary hospitalization reports were used to identify COVID-19–associated hospitalizations. A lifetable method was used to calculate hazard rates (average daily cases during a 7-day interval or hospitalizations over a 14-day interval), hazard ratios, and 95% CIs for each cohort. Rates were age-adjusted to 2000 U.S. Census data using direct standardization.^¶¶^ Supplementary analyses stratified case rates by timing of previous diagnoses and primary series vaccine product. SAS (version 9.4; SAS Institute) and R (version 4.0.4; The R Foundation) were used to conduct all analyses. Institutional review boards (IRBs) in both states determined this surveillance activity to be necessary for public health work, and therefore, it did not require IRB review.

Approximately three quarters of adults from California (71.2%) and New York (72.2%) included in this analysis were vaccinated and did not have a previous COVID-19 diagnosis; however, 18.0% of California residents and 18.4% of New York residents were unvaccinated with no previous COVID-19 diagnosis (Table 1). In both states, 4.5% of persons were vaccinated and had a previous COVID-19 diagnosis; 6.3% in California and 4.9% in New York were unvaccinated with a previous diagnosis. Among 1,108,600 incident COVID-19 cases in these cohorts (752,781 in California and 355,819 in New York), the median intervals from vaccination or previous COVID-19 diagnosis to incident diagnosis were slightly shorter in California (138–150 days) than in New York (162–171 days).

**TABLE 1:** Cohort sizes and cohort-specific incident laboratory-confirmed COVID-19 cases in California (N = 752,781) and New York (N = 355,819) and hospitalizations in California (N = 56,177) — May 30–November 20, 2021

State/Vaccination and diagnosis status*^,†^	No. of persons in each cohort (%)	Incident laboratory-confirmed COVID-19 cases			Incident COVID-19 hospitalizations**
		No. (cumulative incidence)^§,¶^	Median (IQR) interval from vaccination to positive test, days	Median (IQR) interval from previous diagnosis to positive test, days	No. (cumulative incidence)^§,¶^
**California**					
**Vaccinated**					
Previous COVID-19 diagnosis	968,167 (4.5)	3,471 (3.6)	138 (95–181)	262 (218–322)	273 (0.3)
No previous diagnosis	15,484,235 (71.2)	240,045 (15.5)	150 (112–189)	NA	10,737 (0.7)
**Unvaccinated**					
Previous COVID-19 diagnosis	1,370,782 (6.3)	6,805 (5.0)	NA	277 (229–356)	378 (0.3)
No previous diagnosis	3,911,146 (18.0)	502,460 (128.5)	NA	NA	44,789 (11.5)
**New York**					
**Vaccinated**					
Previous COVID-19 diagnosis	485,649 (4.5)	2,355 (4.9)	162 (118–201)	276 (227–348)	NA
No previous diagnosis	7,809,968 (72.2)	142,388 (18.2)	171 (133–203)	NA	NA
**Unvaccinated**					
Previous COVID-19 diagnosis	527,140 (4.9)	3,250 (6.2)	NA	295 (242–427)	NA
No previous diagnosis	1,993,709 (18.4)	207,826 (104.2)	NA	NA	NA

**Abbreviations:** NA = not applicable; NAAT = nucleic acid amplification test.

* Statewide immunization databases in California are the California Immunization Registry, Regional Immunization Data Exchange, and San Diego Immunization Registry, and the laboratory system is the California COVID Reporting System; in New York, Immunization Information Systems include Citywide Immunization Registry and the New York State Immunization Information System; the laboratory system is the Electronic Clinical Laboratory Reporting System. California data were matched between the immunization and case registries using a probabilistic algorithm with exact match for zip code and date of birth and fuzzy match on first name and last name. New York data were matched to the Electronic Clinical Laboratory Reporting System with the use of a deterministic algorithm based on first name, last name, and date of birth. In California, person-level hospitalization data from the California COVID Reporting System and supplemental hospitalization reports were used to identify COVID-19-associated hospitalizations.

^†^ For both classification into cohorts of persons with previous COVID-19 diagnoses and for measuring incident cases, laboratory-confirmed infection was defined as the receipt of a new positive SARS-CoV-2 NAAT or antigen test (both for New York and NAAT only for California) result, but not within 90 days of a previous positive result. Fully vaccinated is defined as having received a second dose of an mRNA COVID-19 vaccine (Pfizer-BioNTech or Moderna) or 1 dose of the Janssen (Johnson & Johnson) vaccine ≥14 days before May 30, 2021. Whereas vaccinated cohorts were directly observed in the immunization information system databases, unvaccinated persons without a previous COVID-19 diagnosis were defined using U.S. Census population estimates minus persons partially or fully vaccinated by December 11, 2021, and unvaccinated persons with a previous laboratory-confirmed infection before May 30, 2021. In California, the California Department of Finance population estimates were used for 2020, and the 2018 CDC National Center for Health Statistics Bridged Race file for census population estimates were used in New York, consistent with other COVID-19 surveillance reporting.

^§^ Cumulative cases per 1,000 persons.

^¶^ These summaries of cumulative incidence are estimated across a period of variability in the epidemic for all cohorts.

** Hospitalization data for New York are not included in this analysis.

Before the Delta variant became predominant in each state’s U.S. Department of Health and Human Services region (June 26 in Region 9 [California] and July 3 in Region 2 [New York]),*** the highest incidence was among unvaccinated persons without a previous COVID-19 diagnosis; during this time, case rates were relatively low among the three groups with either previous infection or vaccination and were lowest among vaccinated persons without a previous COVID-19 diagnosis (Supplementary Figure 1, https://stacks.cdc.gov/view/cdc/113253) (Supplementary Figure 2, https://stacks.cdc.gov/view/cdc/113253). During the week beginning May 30, 2021, compared with COVID-19 case rates among unvaccinated persons without a previous COVID-19 diagnosis, COVID-19 case rates were 19.9-fold (California) and 18.4-fold (New York) lower among vaccinated persons without a previous diagnosis; rates were 7.2-fold (California) and 9.9-fold (New York) lower among unvaccinated persons with a previous COVID-19 diagnosis and 9.6-fold (California) and 8.5-fold (New York) lower among vaccinated persons with a previous COVID-19 diagnosis (Table 2).

**TABLE 2:** Hazard ratios for incident laboratory-confirmed COVID-19 cases — New York and California and hospitalizations* — California, May 30–November 20, 2021

State and date range	Hazard ratio (95% CI)^†^				
	Unvaccinated, no previous COVID-19 diagnosis versus			Vaccinated, no previous COVID-19 diagnosis versus	
	Vaccinated, no previous COVID-19 diagnosis	Unvaccinated, previous COVID-19 diagnosis	Vaccinated, previous COVID-19 diagnosis	Unvaccinated, previous COVID-19 diagnosis	Vaccinated, previous COVID-19 diagnosis
**Cases, California**					
May 30–Jun 5	20.9 (18.9–22.9)	8.2 (6.6–9.9)	10.6 (8.1–13.2)	0.4 (0.3–0.5)	0.5 (0.4–0.6)
Jun 6–12	17.9 (16.2–19.5)	8.6 (6.8–10.4)	10.5 (7.9–13.0)	0.5 (0.4–0.6)	0.6 (0.4–0.7)
Jun 13–19	16.0 (14.7–17.4)	10.8 (8.5–13.2)	10.6 (8.2–13.1)	0.7 (0.5–0.8)	0.7 (0.5–0.8)
Jun 20–26	12.3 (11.4–13.1)	14.5 (11.2–17.8)	17.3 (12.8–21.8)	1.2 (0.9–1.5)	1.4 (1.0–1.8)
Jun 27–Jul 3	9.7 (9.2–10.2)	16.6 (13.5–19.7)	20.9 (16.0–25.8)	1.7 (1.4–2.0)	2.2 (1.6–2.7)
Jul 4–10	8.7 (8.4–9.0)	24.0 (20.1–28.0)	29.3 (23.1–35.6)	2.8 (2.3–3.2)	3.4 (2.6–4.1)
Jul 11–17	7.8 (7.5–8.0)	29.0 (25.0–32.9)	33.4 (27.3–39.4)	3.7 (3.2–4.2)	4.3 (3.5–5.1)
Jul 18–24	7.4 (7.2–7.6)	31.8 (28.1–35.6)	35.2 (29.8–40.6)	4.3 (3.8–4.8)	4.7 (4.0–5.5)
Jul 25–31	7.5 (7.4–7.7)	26.5 (24.1–29.0)	38.6 (33.3–43.9)	3.5 (3.2–3.8)	5.1 (4.4–5.8)
Aug 1–7	7.8 (7.6–7.9)	32.6 (29.5–35.6)	42.2 (36.7–47.7)	4.2 (3.8–4.6)	5.4 (4.7–6.1)
Aug 8–14	8.1 (7.9–8.2)	33.4 (30.4–36.5)	43.1 (37.6–48.6)	4.1 (3.8–4.5)	5.3 (4.7–6.0)
Aug 15–21	8.4 (8.3–8.6)	31.3 (28.5–34.1)	42.0 (36.7–47.3)	3.7 (3.4–4.0)	5.0 (4.3–5.6)
Aug 22–28	8.4 (8.3–8.6)	31.3 (28.4–34.3)	41.0 (35.5–46.5)	3.7 (3.4–4.1)	4.9 (4.2–5.5)
Aug 29–Sep 4	8.5 (8.3–8.6)	31.2 (28.1–34.3)	42.0 (36.1–48.0)	3.7 (3.3–4.1)	5.0 (4.3–5.7)
Sep 5–11	8.3 (8.1–8.5)	35.0 (31.0–39.0)	48.0 (40.2–55.9)	4.2 (3.7–4.7)	5.8 (4.8–6.7)
Sep 12–18	8.4 (8.2–8.6)	33.8 (29.9–37.8)	48.0 (39.8–56.2)	4.0 (3.6–4.5)	5.7 (4.7–6.7)
Sep 19–25	8.0 (7.8–8.2)	27.0 (23.8–30.1)	37.8 (31.5–44.1)	3.4 (3.0–3.8)	4.7 (4.0–5.5)
Sep 26–Oct 2	7.7 (7.5–7.9)	28.6 (24.9–32.2)	34.8 (28.9–40.7)	3.7 (3.2–4.2)	4.5 (3.7–5.3)
Oct 3–9	7.2 (7.0–7.4)	30.0 (26.0–34.1)	33.5 (28.5–38.6)	4.1 (3.6–4.7)	4.6 (3.9–5.3)
Oct 10–16	7.2 (7.0–7.4)	31.2 (26.8–35.7)	33.9 (27.8–40.0)	4.3 (3.7–5.0)	4.7 (3.9–5.5)
Oct 17–23	7.1 (7.0–7.3)	31.9 (27.6–36.1)	40.7 (33.3–48.1)	4.5 (3.9–5.0)	5.7 (4.7–6.7)
Oct 24–30	7.1 (6.9–7.3)	26.6 (23.3–29.9)	40.1 (32.9–47.3)	3.7 (3.3–4.2)	5.6 (4.6–6.6)
Oct 31–Nov 6	6.8 (6.6–7.0)	33.1 (28.7–37.6)	37.9 (31.0–44.7)	4.9 (4.2–5.5)	5.5 (4.5–6.6)
Nov 7–13	7.1 (6.9–7.3)	30.6 (26.3–35.0)	41.2 (33.0–49.5)	4.3 (3.7–4.9)	5.8 (4.6–7.0)
Nov 14–20	7.3 (7.0–7.5)	25.4 (21.4–29.3)	32.5 (25.5–39.5)	3.5 (2.9–4.0)	4.5 (3.5–5.5)
**Cases, New York**					
May 30–Jun 5	19.4 (16.9–21.8)	10.9 (7.5–14.3)	9.5 (6.7–12.4)	0.6 (0.4–0.7)	0.5 (0.3–0.7)
Jun 6–12	15.2 (13.2–17.2)	8.0 (5.5–10.6)	10.4 (6.6–14.3)	0.5 (0.4–0.7)	0.7 (0.4–0.9)
Jun 13–19	12.8 (11–14.5)	8.2 (5.3–11.2)	5.4 (3.7–7.0)	0.6 (0.4–0.9)	0.4 (0.3–0.6)
Jun 20–26	10.1 (8.8–11.4)	7.9 (5.1–10.7)	6.0 (4.0–8.0)	0.8 (0.5–1.1)	0.6 (0.4–0.8)
Jun 27–Jul 3	7.3 (6.5–8.1)	8.8 (5.8–11.8)	11.2 (6.7–15.7)	1.2 (0.8–1.6)	1.5 (0.9–2.2)
Jul 4–10	6.1 (5.6–6.7)	17.8 (10.6–25.0)	11.5 (7.5–15.6)	2.9 (1.7–4.1)	1.9 (1.2–2.6)
Jul 11–17	4.5 (4.2–4.8)	11.7 (8.5–15.0)	14.7 (9.9–19.6)	2.6 (1.9–3.3)	3.2 (2.2–4.3)
Jul 18–24	4.7 (4.5–5.0)	21.7 (15.6–27.8)	14.1 (10.5–17.7)	4.6 (3.3–5.9)	3.0 (2.2–3.8)
Jul 25–31	5.1 (4.9–5.3)	16.1 (13.1–19.2)	18.3 (14.1–22.6)	3.2 (2.6–3.8)	3.6 (2.8–4.4)
Aug 1–7	5.3 (5.2–5.5)	19.2 (15.9–22.6)	18.3 (14.7–21.9)	3.6 (3.0–4.2)	3.4 (2.7–4.1)
Aug 8–14	5.3 (5.2–5.5)	16.2 (13.8–18.6)	19.2 (15.6–22.7)	3.0 (2.6–3.5)	3.6 (2.9–4.3)
Aug 15–21	5.5 (5.3–5.7)	19.5 (16.5–22.6)	22.7 (18.4–26.9)	3.6 (3.0–4.1)	4.1 (3.4–4.9)
Aug 22–28	5.4 (5.2–5.6)	19.2 (16.4–22.1)	26.5 (21.2–31.8)	3.6 (3.0–4.1)	4.9 (3.9–5.9)
Aug 29–Sep 4	5.5 (5.3–5.6)	17.9 (15.3–20.5)	20.9 (17.2–24.6)	3.3 (2.8–3.8)	3.8 (3.1–4.5)
Sep 5–11	5.4 (5.2–5.5)	18.9 (16.1–21.6)	22.3 (18.3–26.4)	3.5 (3.0–4.0)	4.2 (3.4–4.9)
Sep 12–18	5.8 (5.6–5.9)	15.0 (13.1–16.9)	23.2 (19.1–27.4)	2.6 (2.3–2.9)	4.0 (3.3–4.8)
Sep 19–25	5.6 (5.4–5.7)	15.4 (13.3–17.5)	23.8 (19.3–28.3)	2.8 (2.4–3.1)	4.3 (3.5–5.1)
Sep 26–Oct 2	5.4 (5.2–5.5)	18.4 (15.5–21.2)	24.2 (19.3–29.1)	3.4 (2.9–4.0)	4.5 (3.6–5.4)
Oct 3–9	5.5 (5.3–5.7)	15.7 (13.6–17.9)	20.8 (17.2–24.5)	2.9 (2.5–3.3)	3.8 (3.1–4.4)
Oct 10–16	5.5 (5.3–5.6)	17.2 (14.7–19.8)	25.9 (20.6–31.1)	3.2 (2.7–3.6)	4.7 (3.8–5.7)
Oct 17–23	5.4 (5.2–5.6)	18.9 (15.7–22.1)	27.6 (21.2–34.0)	3.5 (2.9–4.1)	5.1 (3.9–6.3)
Oct 24–30	5.2 (5.0–5.4)	21.0 (17.2–24.7)	25.9 (20.2–31.6)	4.0 (3.3–4.7)	5.0 (3.9–6.1)
Oct 31–Nov 6	4.8 (4.6–4.9)	17.3 (14.7–20.0)	20.1 (16.3–23.8)	3.6 (3.1–4.2)	4.2 (3.4–5.0)
Nov 7–13	4.8 (4.7–4.9)	23.9 (20.1–27.6)	24.5 (20.1–28.9)	5.0 (4.2–5.8)	5.1 (4.2–6.1)
Nov 14–20	4.8 (4.6–4.9)	22.6 (19.4–25.7)	23.0 (19.3–26.6)	4.7 (4.1–5.4)	4.8 (4.1–5.6)
**Hospitalizations, California**					
May 30–Jun 12	29.8 (23.5–36.1)	3.7 (2.5–5.0)	7.2 (4.2–10.1)	0.1 (0.1–0.2)	0.2 (0.1–0.3)
Jun 13–26	28.7 (23.4–34.0)	7.0 (4.3–9.7)	8.1 (5.0–11.3)	0.2 (0.1–0.3)	0.3 (0.2–0.4)
Jun 27–10	30.1 (26.1–34.0)	16.4 (10.0–22.8)	16.0 (10.0–22.1)	0.5 (0.3–0.8)	0.5 (0.3–0.7)
Jul 11–24	25.8 (23.7–28.0)	45.0 (27.6–62.4)	41.5 (25.2–57.8)	1.7 (1.1–2.4)	1.6 (1.0–2.2)
Jul 25–Aug 7	28.8 (27.1–30.6)	41.7 (29.2–54.1)	72.9 (44.4–101.4)	1.4 (1.0–1.9)	2.5 (1.5–3.5)
Aug 8–21	29.7 (28.0–31.4)	49.0 (35.0–62.9)	64.0 (43.0–85.1)	1.6 (1.2–2.1)	2.2 (1.4–2.9)
Aug 22–Sep 4	29.1 (27.4–30.8)	62.4 (41.4–83.3)	63.9 (42.2–85.5)	2.1 (1.4–2.9)	2.2 (1.4–2.9)
Sep 5–18	26.3 (24.6–28.1)	74.4 (40.9–107.9)	96.4 (48.3–144.4)	2.8 (1.5–4.1)	3.7 (1.8–5.5)
Sep 19–Oct 2	25.0 (23.1–26.9)	61.9 (34.5–89.3)	99.4 (43.8–155.0)	2.5 (1.4–3.6)	4.0 (1.7–6.2)
Oct 3–16	20.8 (19.2–22.4)	56.3 (28.3–84.3)	58.5 (30.2–86.8)	2.7 (1.4–4.1)	2.8 (1.4–4.2)
Oct 17–30	21.5 (19.9–23.0)	56.5 (31.5–81.5)	92.1 (39.1–145.1)	2.6 (1.5–3.8)	4.3 (1.8–6.8)
Oct 31–Nov 13	22.7 (20.8–24.6)	70.7 (32.0–109.4)	86.1 (34.2–138.1)	3.1 (1.4–4.8)	3.8 (1.5–6.1)

* Life tables estimated at 7-day intervals for cases and 14-day intervals for hospitalizations.

^†^ Hazard ratios and 95% CIs reported in this table differ numerically from presentation of corresponding results in the text as “X-fold lower” rates (i.e., a hazard rate of 1.0 is zero-fold lower). For example, a hazard ratio of 20.9 (95% CI = 18.9–22.9) for those “Unvaccinated–no previous COVID-19 diagnosis” versus “Vaccinated, no previous COVID-19 diagnosis” is equivalent to a 19.9-fold lower (95% CI = 17.9–21.9) rate for those “Vaccinated, no previous COVID-19 diagnosis” relative to those “Unvaccinated, no previous COVID-19 diagnosis.”

As the Delta variant prevalence increased to >95% (97% in Region 9 and 98% in Region 2 on August 1), rates increased more rapidly among the vaccinated group with no previous COVID-19 diagnosis than among both the vaccinated and unvaccinated groups with a previous COVID-19 diagnosis (Supplementary Figure 1, https://stacks.cdc.gov/view/cdc/113253) (Supplementary Figure 2, https://stacks.cdc.gov/view/cdc/113253). For example, during the week of October 3, compared with rates among unvaccinated persons without a previous COVID-19 diagnosis, rates among vaccinated persons without a previous diagnosis were 6.2-fold lower (95% CI = 6.0–6.4) in California and 4.5-fold lower (95% CI = 4.3–4.7) in New York (Table 2). Further, rates among unvaccinated persons with a previous COVID-19 diagnosis were 29-fold lower (95% CI = 25.0–33.1) than rates among unvaccinated persons without a previous COVID-19 diagnosis in California and 14.7-fold lower (95% CI = 12.6–16.9) in New York. Rates among vaccinated persons who had had COVID-19 were 32.5-fold lower (95% CI = 27.5–37.6) than rates among unvaccinated persons without a previous COVID-19 diagnosis in California and 19.8-fold lower (95% CI = 16.2–23.5) in New York. Rates among vaccinated persons without a previous COVID-19 diagnosis were consistently higher than rates among unvaccinated persons with a history of COVID-19 (3.1-fold higher [95% CI = 2.6–3.7] in California and 1.9-fold higher [95% CI = 1.5–2.3] in New York) and rates among vaccinated persons with a history of COVID-19 (3.6-fold higher [95% CI = 2.9–4.3] in California and 2.8-fold higher [95% CI = 2.1–3.4] in New York).

**FIGURE:**
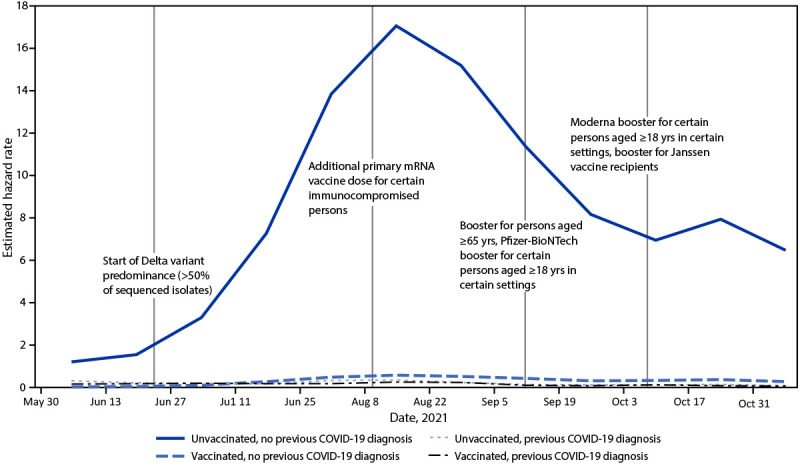
Incident laboratory-confirmed COVID-19-associated hospitalizations among immunologic cohorts defined by vaccination and previous diagnosis histories — California, May 30–November 13, 2021*^,†^ * The SARS-CoV-2 Delta variant exceeded 50% of sequences in U.S. Department of Health and Human Services Region 9 (containing California) during the week of June 26. https://covid.cdc.gov/covid-data-tracker/#variant-proportions † Estimated hazard rate is laboratory-confirmed COVID-19-associated hospitalizations per 100,000 person-days visualized at midpoint of each reporting interval.

COVID-19 hospitalization rates in California were always highest among unvaccinated persons without a previous COVID-19 diagnosis (Table 2) (Figure). In the pre-Delta period during June 13–June 26, for example, compared with hospitalization rates among unvaccinated persons without a previous COVID-19 diagnosis, hospitalization rates were 27.7-fold lower (95% CI = 22.4–33.0) among vaccinated persons without a previous COVID-19 diagnosis, 6.0-fold lower (95% CI = 3.3–8.7) among unvaccinated persons with a previous COVID-19 diagnosis, and 7.1-fold lower (95% CI = 4.0–10.3) among vaccinated persons with a previous COVID-19 diagnosis. However, this pattern also shifted as the Delta variant became predominant. During October 3–16, compared with hospitalization rates among unvaccinated persons without a previous COVID-19 diagnosis, hospitalization rates were 19.8-fold lower (95% CI = 18.2–21.4) among vaccinated persons without a previous COVID-19 diagnosis, 55.3-fold lower (95% CI = 27.3–83.3) among unvaccinated persons with a previous COVID-19 diagnosis, and 57.5-fold lower (95% CI = 29.2–85.8) among vaccinated persons with a previous COVID-19 diagnosis.

Among the two cohorts with a previous COVID-19 diagnosis, no consistent incidence gradient by time since the previous diagnosis was observed (Supplementary Figure 3, https://stacks.cdc.gov/view/cdc/113253). When the vaccinated cohorts were stratified by the vaccine product received, among vaccinated persons without a previous COVID-19 diagnosis, the highest incidences were observed among persons receiving the Janssen (Johnson & Johnson), followed by Pfizer-BioNTech, then Moderna vaccines (Supplementary Figure 4, https://stacks.cdc.gov/view/cdc/113253). No pattern by product was observed among vaccinated persons with a previous COVID-19 diagnosis.

## Discussion

This analysis integrated laboratory testing, hospitalization surveillance, and immunization registry data in two large states during May–November 2021, before widespread circulation of the SARS-CoV-2 Omicron variant and before most persons had received additional or booster COVID-19 vaccine doses to protect against waning immunity. Rate estimates from the analysis describe different experiences stratified by COVID-19 vaccination status and previous COVID-19 diagnosis and during times when different SARS-CoV-2 variants predominated. Case rates were initially lowest among vaccinated persons without a previous COVID-19 diagnosis; however, after emergence of the Delta variant and over the course of time, incidence increased sharply in this group, but only slightly among both vaccinated and unvaccinated persons with previously diagnosed COVID-19 (*6*). Across the entire study period, persons with vaccine- and infection-derived immunity had much lower rates of hospitalization compared with those in unvaccinated persons. These results suggest that vaccination protects against COVID-19 and related hospitalization and that surviving a previous infection protects against a reinfection. Importantly, infection-derived protection was greater after the highly transmissible Delta variant became predominant, coinciding with early declining of vaccine-induced immunity in many persons (*5*). Similar data accounting for booster doses and as new variants, including Omicron, circulate will need to be assessed.

The understanding and epidemiology of COVID-19 has shifted substantially over time with the emergence and circulation of new SARS-CoV-2 variants, introduction of vaccines, and changing immunity as a result. Similar to the early period of this study, two previous U.S. studies found more protection from vaccination than from previous infection during periods before Delta predominance (*3*,*7*). As was observed in the present study after July, recent international studies have also demonstrated increased protection in persons with previous infection, with or without vaccination, relative to vaccination alone^†††^^,^^ §§§^ (*4*). This might be due to differential stimulation of the immune response by either exposure type.^¶¶¶^ Whereas French and Israeli population-based studies noted waning protection from previous infection, this was not apparent in the results from this or other large U.K. and U.S. studies**** (*4*,*8*). Further studies are needed to establish duration of protection from previous infection by variant type, severity, and symptomatology, including for the Omicron variant. 

The findings in this report are subject to at least seven limitations. First, analyses were not stratified by time since vaccine receipt, but only by time since previous diagnosis, although earlier studies have examined waning of vaccine-induced immunity (Supplementary Figure 3, https://stacks.cdc.gov/view/cdc/113253) (*2*). Second, persons with undiagnosed infection are misclassified as having no previous COVID-19 diagnosis; however, this misclassification likely results in a conservative bias (i.e., the magnitude of difference in rates would be even larger if misclassified persons were not included among unvaccinated persons without a previous COVID-19 diagnosis). California seroprevalence data during this period indicate that the ratio of actual (presumptive) infections to diagnosed cases among adults was 2.6 (95% CI = 2.2–2.9).^††††^ Further, California only included NAAT results, whereas New York included both NAAT and antigen test results. However, antigen testing made up a smaller percentage of overall testing volume reported in California (7% of cases) compared with New York (25% of cases) during the study period. Neither state included self-tests, which are not easily reportable to public health. State-specific hazard ratios were generally comparable, although differences in rates among unvaccinated persons with a previous COVID-19 diagnosis were noteworthy. Third, potential exists for bias related to unmeasured confounding (e.g., behavioral or geographic differences in exposure risk) and uncertainty in the population size of the unvaccinated group without a previous COVID-19 diagnosis. Persons might be more or less likely to receive testing based on previous diagnosis or vaccination status; however, different trajectories between vaccinated persons with and without a previous COVID-19 diagnosis, and similar findings for cases and hospitalizations, suggest that these biases were minimal. Fourth, this analysis did not include information on the severity of initial infection and does not account for the full range of morbidity and mortality represented by the groups with previous infections. Fifth, this analysis did not ascertain receipt of additional or booster COVID-19 vaccine doses and was conducted before many persons were eligible or had received additional or booster vaccine doses, which have been shown to confer additional protection.^§§§§^ Sixth, some estimates lacked precision because of sample size limitations. Finally, this analysis was conducted before the emergence of the Omicron variant, for which vaccine or infection-derived immunity might be diminished.^¶¶¶¶^ This study offers a surveillance data framework to help evaluate both infections in vaccinated persons and reinfections as new variants continue to emerge.

Vaccination protected against COVID-19 and related hospitalization, and surviving a previous infection protected against a reinfection and related hospitalization during periods of predominantly Alpha and Delta variant transmission, before the emergence of Omicron; evidence suggests decreased protection from both vaccine- and infection-induced immunity against Omicron infections, although additional protection with widespread receipt of booster COVID-19 vaccine doses is expected. Initial infection among unvaccinated persons increases risk for serious illness, hospitalization, long-term sequelae, and death; by November 30, 2021, approximately 130,781 residents of California and New York had died from COVID-19. Thus, vaccination remains the safest and primary strategy to prevent SARS-CoV-2 infections, associated complications, and onward transmission. Primary COVID-19 vaccination, additional doses, and booster doses are recommended by CDC’s Advisory Committee on Immunization Practices to ensure that all eligible persons are up to date with COVID-19 vaccination, which provides the most robust protection against initial infection, severe illness, hospitalization, long-term sequelae, and death.***** Additional recommendations for vaccine doses might be warranted in the future as the virus and immunity levels change.

**Summary**

**What is already known about this topic?**

Data are limited regarding the risks for SARS-CoV-2 infection and hospitalization after COVID-19 vaccination and previous infection.

**What is added by this report?**

During May–November 2021, case and hospitalization rates were highest among persons who were unvaccinated without a previous diagnosis. Before Delta became the predominant variant in June, case rates were higher among persons who survived a previous infection than persons who were vaccinated alone. By early October, persons who survived a previous infection had lower case rates than persons who were vaccinated alone.

**What are the implications for public health practice?**

Although the epidemiology of COVID-19 might change as new variants emerge, vaccination remains the safest strategy for averting future SARS-CoV-2 infections, hospitalizations, long-term sequelae, and death. Primary vaccination, additional doses, and booster doses are recommended for all eligible persons. Additional future recommendations for vaccine doses might be warranted as the virus and immunity levels change.

## Footnotes

*
https://covid.cdc.gov/covid-data-tracker/#cases_deathsper100klast7days

† Statewide immunization databases in California are the California Immunization Registry, Regional Immunization Data Exchange, and San Diego Immunization Registry; the laboratory system is the California COVID Reporting System (CCRS). In New York, immunization information systems include Citywide Immunization Registry and the New York State Immunization Information System; the laboratory system is the Electronic Clinical Laboratory Reporting System (ECLRS). California data were matched between the immunization and case registries using a probabilistic algorithm with exact match for zip code and date of birth and fuzzy match on first name and last name. New York data were matched to the ECLRS with the use of a deterministic algorithm based on first name, last name, and date of birth. In California, person-level hospitalization data from CCRS and supplementary hospitalization reports were used to identify COVID-19–associated hospitalizations.

§ For both classification into cohorts of persons with previous COVID-19 diagnoses and for measuring incident cases, laboratory-confirmed infection was defined as the receipt of a new positive SARS-CoV-2 nucleic acid amplification test (NAAT) or antigen test (both for New York and NAAT only for California) result, but not within 90 days of a previous positive result.

¶ Fully vaccinated with the primary vaccination series is defined as receipt of a second dose of an mRNA COVID-19 vaccine (Pfizer-BioNTech or Moderna) or 1 dose of the Janssen (Johnson & Johnson) vaccine ≥14 days before May 30, 2021.

** Because of the timing of full vaccination, the cohort definitions, and analysis timeframe, this cohort consisted nearly exclusively of persons who had previously received a laboratory-confirmed diagnosis of COVID-19 and later were fully vaccinated (California: 99.9%, New York: 99.7%), as opposed to the reverse order.

†† Whereas vaccinated cohorts were directly observed in the immunization information system databases, unvaccinated persons without a previous COVID-19 diagnosis were defined using U.S. Census population estimates minus the number of persons partially or fully vaccinated by December 11, 2021, and unvaccinated persons with a previous laboratory-confirmed infection before May 30, 2021. In California, the California Department of Finance population estimates were used for 2020, and the 2018 CDC National Center for Health Statistics Bridged Race file for U.S. Census population estimates were used in New York, consistent with other COVID-19 surveillance reporting.

§§ In California, a person-level match was performed to exclude deaths in each cohort before May 30, 2021. In New York, COVID-19 deaths were removed in aggregate from the starting number of unvaccinated persons with a previous COVID-19 diagnosis on May 30, 2021.

¶¶
https://www.cdc.gov/nchs/data/statnt/statnt20.pdf

***
https://covid.cdc.gov/covid-data-tracker/#variant-proportions

†††
https://www.medrxiv.org/content/10.1101/2021.09.12.21263461v1

§§§
https://www.medrxiv.org/content/10.1101/2021.11.29.21267006v1

¶¶¶
https://www.cdc.gov/coronavirus/2019-ncov/science/science-briefs/vaccine-induced-immunity.html#anchor_1635540449320

****
https://www.medrxiv.org/content/10.1101/2021.12.04.21267114v1

††††
https://www.medrxiv.org/content/10.1101/2021.12.09.21267565v1

§§§§
https://covid.cdc.gov/covid-data-tracker/#rates-by-vaccine-status

¶¶¶¶ https://www.medrxiv.org/content/10.1101/2021.12.30.21268565v1; https://www.medrxiv.org/content/10.1101/2022.01.07.22268919v1

*****
https://www.cdc.gov/vaccines/covid-19/clinical-considerations/covid-19-vaccines-us.html
